# Frequency of red blood cell allo-immunization in patients undergoing blood transfusion at the Uganda Cancer Institute

**DOI:** 10.4314/ahs.v23i4.39

**Published:** 2023-12

**Authors:** Clement D Okello, Andrew W Shih, Martin Nabwana, Noah Kiwanuka, Nancy Heddle, Harriet Mayanja-Kizza, Jackson Orem

**Affiliations:** 1 Uganda Cancer Institute; 2 Department of Pathology and Laboratory Medicine, University of British Columbia, Vancouver, BC, Canada; 3 Makerere University – Johns Hopkins University Research Collaboration; 4 School of Public Health, College of Health Sciences, Makerere University, Uganda; 5 McMaster Centre for Transfusion Research, Department of Medicine, McMaster University, Hamilton ON, Canada; 6 Department of Medicine, College of Health Sciences, Makerere University, Uganda

**Keywords:** Allo-immunization, blood, transfusion, Cancer, Uganda

## Abstract

**Background:**

There is limited data on red blood cell (RBC) alloimmunization in patients with cancer in sub-Saharan Africa (SSA). We examined the frequency of RBC alloimmunization in transfused patients with cancers in Uganda.

**Methods:**

A randomized control trial was conducted on participants at the Uganda Cancer Institute. Eligible participants were age ≥15 years and required blood transfusion. Participants were randomized to receive either leucoreduced or non-leucoreduced blood transfusion. Participants' plasma samples were screened for RBC alloantibodies at enrolment and 3-4 weeks after blood transfusion using a 2-cell panel of reagent group O RBCs using the tube method. Antibody identification was performed using a 10-cell panels of reagent RBCs. Participants were considered alloimmunized if antibodies to RBC antigens were identified.

**Results:**

A total of 277 participants were randomized (leucoreduced blood, n=137; non-leucoreduced blood, n=140). Overall, the most represented diagnoses were gynaecological cancers (n=88, 31.8%), acute leukaemia (n=35, 12.6%), and gastrointestinal cancers (n=25, 9.0%). Concomitant HIV infection was present in 26 (9.4%) participants. Most participants received <5 units of blood during the study. No study participant developed allo-antibodies.

**Conclusion:**

There was no RBC alloimmunization in participants with cancers. Routine RBC allo-antibody screening in all patients with cancer in SSA requires further research.

## Background

Blood transfusion is a cornerstone of treatment in every dimension of medicine. In sub-Saharan Africa (SSA), blood transfusion is mainly used in the treatment of patients with cancer (33.5%), pregnancy (12.4%) and sickle cell disease (6.9%) 1. In a recent retrospective study involving 2,012 patients transfused with whole blood in Uganda, cancer was the most represented diagnosis, accounting for 17.7% of the cases 2.

Despite extensive pre-transfusion testing and mitigation strategies, blood transfusion is still associated with numerous complications 3. Red blood cell (RBC) antibody formation against the transfused foreign RBC antigens, also known as alloimmunization, is one major complication in transfused patients 4 that commonly occurs from 24 hours up to 21 days after a transfusion 5. Although mortality resulting directly from alloimmunization is relatively rare 6, more common transfusion associated complications from RBC alloimmunization include challenges in locating compatible blood for alloimmunized persons, delayed hemolytic transfusion reactions, and rarely, acute hemolytic transfusion reactions 4. Risk factors for alloimmunization are multifactorial, including recipient's sex, age, age at first transfusion, age of RBCs transfused, number of blood transfusions, RBC antigen disparity, race, and patients' genetic and inflammatory status 4, 7-13.

The frequency of RBC alloimmunization has been reported in up to 76% of multiply transfused patients with sickle cell disease (SCD) in Europe 7, 14. In SSA, the frequency of RBC alloimmunization range from 2.6 - 18.8 %, with the highest levels in patients with SCD 15-18. However, there is limited data on the frequency of RBC alloimmunization in patients with cancer in SSA. Transfusion with leucoreduced blood was noted to significantly reduce the incidence of RBC alloantibodies in patients with cancer in a hospital in New York City, USA 19. In this prospective study with a historical control, the rate of RBC alloimmunization in patients transfused with non-leucoreduced blood was 8.2% and 2.8% in patients transfused with only leucoreduced blood (p = 0.02) 19.

To our knowledge, there is no published randomized control trial (RCT) comparing the rate of RBC alloimmunization in patients transfused with leucoreduced and non-leucoreduced blood. The current study was a secondary objective of an RCT in recipients of leucoreduced and recipients of non-leucoreduced whole blood transfusion in Uganda.

## Methods

### Study design

An open-label RCT comparing leucoreduced blood transfusion to non-leucoreduced blood transfusion was performed, with the primary outcome of this study being RBC alloimmunization. We hypothesized that RBC alloimmunization rates in patients receiving transfusions would be lower in the leucoreduced Participants were randomized using permuted block randomization. Block sizes of 6 were used with a 1:1 allocation ratio across the leucoreduced and non-leucoreduced blood transfusion arms. The randomization numbers were generated by a statistician using a computer-based random number generation sequence. The statistician then informed the principal investigator of the randomization numbers with their corresponding treatment assignment in groups of five each time until the numbers were exhausted.

### Study setting

The study was conducted at the Uganda Cancer Institute (UCI), the tertiary cancer treatment facility located in Kampala, the capital city of Uganda. Patients with various types of cancers including solid tumours, and haematological cancers are treated at the UCI. Blood transfusion at the UCI is prescribed by a physician, and follows the prescription guidelines from the Ugandan Ministry of Health which recommends blood transfusion based on the clinical condition of the patient, and especially when the haemoglobin level is <7 g/dL or <6g /dL for patients with sickle cell anaemia.

Blood components are prepared and provided by the Uganda Blood Transfusion Services (UBTS), Nakasero-Kampala. Prior to delivery to the hospitals, all blood is serologically tested and released only when found negative for human immunodeficiency virus, hepatitis B and C, and syphilis. Non-leucoreduced whole blood transfusion was the current standard of care in Uganda while undertaking this study. Whole blood units provided by UBTS for transfusion were preserved in citrate phosphate dextrose adenine (CPDA-1) and kept under refrigerated storage at 1 – 6°C. Leucocyte filtration of whole blood was unique to this study, and was performed at the UBTS using commercially acquired equipment (LEUCOLAB LCG4b, Macopharma-Rue Lorthiosis, Mouvaux-France) in accordance with the product manual. Leucocyte filtration was performed within 72 hours of blood collection. Samples from randomly selected leucoreduced whole blood bags were analyzed for quality control and achieved a residual leucocyte counts of <1x10^6^/unit.

At the hospital, pre-transfusion testing consisted of recipient's ABO and Rhesus D blood typing, and a room temperature immediate spin cross-match – all performed using the tile method. For this study, red blood cell allo-antibody screening was done at enrolment and at 3-4 weeks after enrolment. Of note, red blood cell allo-antibody screening is not routinely performed prior to blood transfusion.

### Study participants

Patients of ≥ 15 years of age, who needed blood transfusion as judged by the primary care team and were admitted to the Uganda Cancer Institute were eligible for enrolment in the study. Patients were identified from the transfusion laboratory based on the submitted request for blood transfusion. Those who met the study eligibility and provided informed consent were enrolled and were followed for 3 – 4 weeks since RBC alloimmunization commonly occurs up to 3 weeks after a blood transfusion 5. If a patient had a positive first antibody screen at the time of assessment for study eligibility, they were excluded from the randomization to either receive leucoreduced or non-leucoreduced blood. However, this data was included in our analyses to determine the prevalence of RBC alloimmunization as described further below.

### Laboratory investigation

Blood samples from the participants were collected in the ethylenediaminetetraacetic acid (EDTA) tubes and centrifuged. The suspended plasma was used for RBC allo-antibody screening. Plasma samples were screened for the presence of RBC alloantibodies by use of a 2-cell panel of reagent group O RBCs (IMMUCOR, INC. Norcross, GA USA) using the tube method. In the indirect antiglobulin test, a polyspecific anti-human globulin (anti-IgG and -C3d) were used. IgG sensitized cells (check cells) were added to all negative indirect antiglobulin tests. At least a grade 2 reaction was expected following the addition of check cells for the test to be considered valid, otherwise they were repeated. When the antibody screening was positive, antibody identification was performed by testing the same plasma samples with a 10-cell panels of reagent RBCs of selected phenotypes (IMMUCOR, INC. Norcross, GA USA). Patients were considered to be alloimmunized if antibodies to one or more RBC antigens were identified. Positive test results were immediately communicated to the treating physician to guide patient management.

### Data Collection

All data were manually recorded into a standardized data collection form. Data collected included demographic information, diagnoses, baseline performance score using the Eastern Cooperative Oncology Group (ECOG) criteria, comorbidities, history of previous pregnancies, number of previous blood transfusion and transfusion received during the study, rhesus blood group type, and RBC allo-antibody status at baseline and at the end of study follow up. Transfusion with non-study related blood products were captured. Platelets were not leucoreduced. Data collected were verified for completeness and accuracy by the principal investigator. All patients' information was anonymized after data verification. Verified data were coded, and entered into a database using Epidata version 3.1 (Epidata association, Denmark) and cleaned to ensure accuracy before exporting to STATA Version 15 (StataCorp, USA) for analysis. Data were locked in a secure place and the database was kept in a computer secured with a password. Study approvals were obtained from the Makerere University School of Medicine Research Ethics Committee (Ref. 2017-106) and the Uganda National Council for Science and Technology (Ref. HS 2705). The RCT was registered at the Uganda National Drug Authority (CTA-0137).

### Statistical analysis

Demographic and clinical data were reported using descriptive statistics with mean and standard deviation for parametric data and median and IQR for nonparametric data. The prevalence of RBC alloimmunization was expressed as a proportion of participants with a positive baseline RBC allo-antibodies to the total number of persons screened at baseline. The incidence of RBC alloimmunization was expressed as a proportion of participants with positive RBC allo-antibody after the 3-4 weeks of study follow up when they had negative RBC allo-antibody test at the study enrolment.

## Results

### Baseline characteristics

**Figure 1:**
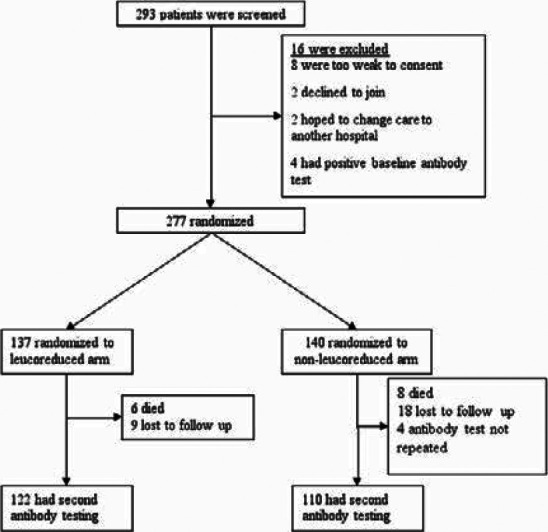
Screening, randomization & follow up

Two hundred ninety-three participants were screened, of whom 277 participants were randomized (137 on the leucoreduced blood transfusion arm and 140 on the non-leucoreduced blood transfusion arm); 16 participants did not meet the eligibility criteria as shown in Figure 1.

The characteristics of the participants at the time of enrolment were similar for both study arms as summarized in Table 1.

**Table 1:** Baseline characteristics

Characteristics	Number (%), Overall	Leucoreduced arm	Non-leucoreduced arm	p-value
**Age (IQR)**	40 (30, 52)	42 (33, 55)	39 (26.5, 50.0)	0.209
**Sex**				
M	109 (39.35)	46 (33.58)	63 (45)	
F	168 (60.65)	91 (66.42)	77 (55)	0.05
**ECOG**				
1	63 (22.74)	26 (18.98)	37 (26.43)	
2	152 (54.87)	84 (61.31)	68 (48.57)	
3	51 (18.41)	23 (16.79)	28 (20)	
4	11 (3.97)	4 (2.92)	7 (5)	0.18
**Pregnancy(ies), n=145**				
1	12 (8.3)	6 (7.59)	6 (9.09)	
2-4	61 (42.1)	38 (48.1)	23 (34.85)	
>5	72 (49.6)	35 (44.3)	37 (56.06)	0.27
**ABO blood group type**				
A	63 (22.74)	28 (20.44)	35 (25)	
B	57 (20.58)	35 (25.55)	22 (15.71)	
AB	13 (4.69)	3 (2.19)	10 (7.14)	
O	144 (51.99)	71 (51.82)	73 (50.54)	0.06
**Rhesus blood group type**				
Negative	3 (1.08)	1 (0.73)	2 (1.43)	
Positive	274 (98.92)	136 (99.27)	138 (98.57)	0.57
**Previous blood transfusion**				
**Whole blood**				
1	33 (30.28)	20 (36.36)	13 (24.07)	
2-4	40 (36.7)	18 (32.73)	22 (40.74)	
> 5	36 (33.03)	17 (30.91)	19 (35.19)	0.37
**Packed cells**				
1	21 (36.21)	10 (45.45)	11 (30.56)	
2-4	20 (34.48)	6 (27.27)	14 (38.89)	
> 5	17 (29.31)	6 (27.27)	11 (30.56)	0.49
**Platelets**				
1	1 (6.25)	1 (14.29)	0	
2-4	5 (31.25)	2 (28.14)	3 (33.33)	
> 5	10 (62.5)	4 (57.14)	6 (66.67)	0.50

### Diagnosis

Both study arms had comparable distribution of diagnoses. Overall, the most represented diagnoses were gynaecological cancers (n=88, 31.8%), acute leukemia (n=35, 12.6%), and gastrointestinal cancers (n=25, 9.0%), Table 2. Twenty-six (9.4%) participants had concomitant HIV infection.

**Table 2:** Participant diagnosis

Diagnosis	Number (%), Overall	Leucoreduced arm	Non-leucoreduced arm	P-value
Gynaecological Cancers	88 (31.8)	49 (35.8)	39 (27.9)	0.376
Acute Leukemia	35 (12.6)	17 (12.4)	18 (12.9)	
GI Cancers	25 (9.0)	12 (8.8)	13 (9.3)	
Urological Cancers	22 (7.9)	10 (7.3)	12 (8.6)	
Breast Cancer	20 (7.2)	13 (9.5)	7 (5)	
Skin and Sarcomas	19 (6.9)	8 (5.8)	11 (7.9)	
Lymphoma	16 (5.8)	5 (3.6)	11 (7.9)	
Non-Cancers	9 (3.2)	3 (2.2)	6 (4.3)	
Chronic Leukemias	9 (3.2)	3 (2.2)	6 (4.3)	
Kaposi Sarcoma	8 (2.9)	4 (2.9)	4 (2.9)	
Multiple Myeloma	7 (2.5)	6 (4.4)	1 (0.7)	
Head and Neck	6 (2.2)	3 (2.2)	3 (2.1)	
Other Cancers	6 (2.2)	3 (2.2)	3 (2.1)	
Brain tumors	4 (1.4)	1 (0.7)	3 (2.1)	
Bone Tumors	3 (1.1)	0	3 (2.1)	

### Blood transfusion received during the study

Most participants in both study arms received 1 – 4 units of blood transfusion, Table 3. Four participants in the leucoreduced arm and 5 participants in the non-leucoreduced arm were also transfused with RhD-matched platelets; of these, three of the four participants in the leucoreduced arm and all the five participants in the non-leucoreduced arm were transfused with 10 or more units of RhD-matched platelets during the study.

**Table 3:** Number of transfusions received during the 30-day study period

Number of blood units transfused	Leucoreduced blood, n=137 (49.5%)	Non-leucoreduced blood, n=140 (50.5%)
Repeat antibody test done		
1-4 units	111 (40.1)	95 (34.3)
5-9 units	11* (4.0)	13 (4.7)
≥10 units	0	2 (0.7)
Repeat antibody test not done	15 (5.4)	30 (10.8)

* Four participants also received non-leucoreduced blood

### Red blood cell allo-antibody

Four of the 293 screened participants (1.4%) had positive test during the initial RBC allo-antibody screen test. However, at identification, these participants had uniform reactions across all panel cells at the immediate spin phase (room temperature), suggestive of autoantibodies. Further techniques including antibody elution and/or autoadsorption were not performed on the positive samples. The characteristics of the four participants with positive RBC allo-antibody at screening is illustrated in Table 4. Of the 277 participants with negative RBC allo-antibody test at baseline, 44% in the leucoreduced arm and 40% in the non-leucoreduced arm had repeat RBC allo-antibody testing at the end of followp. None of these participants tested positive for the RBC allo-antibody at the second testing.

**Table 4:** Characteristics of participants with positive antibody results at screening

Age	Sex	Number of Pregnancy(ies)	Number of prior blood transfusion	Diagnosis	Chemotherapy exposure
53	F	3	7	CLL/SLL	Chlorambucil, Prednisolone
57	M	N/A	2	Urothelial bladder cancer	Gemcitabine, Carboplatin
41	F	3	4	Cervical cancer	Cisplatin
70	M	N/A	2	CLL	Chlorambucil, Prednisolone

**Note:** CLL – Chronic lymphocytic leukemia; SLL – Small lymphocytic lymphoma; F – Female; M – Male; N/A- Not Applicable.

## Discussion

This study was conducted on cancer patients who had blood transfusion in a sub-Sahara African (SSA) setting, where we observed no RBC alloimmunization in both the leucoreduced and non-leucoreduced arms of the study population. The RBC alloimmunization in this study is lower than expected despite a high proportion of female patients (60.7%) with a prior pregnancy (52.3% of females in our study). Female sex is a known risk factor for RBC alloimmunization 8 probably due to the transfer of foetal blood cells into the maternal circulation as a complication during pregnancy. In addition, a number of our study participants had multiple exposures to blood transfusion prior to (66%) and during the study period. Only presumed cold autoantibodies were recorded in 4/293 (1.4%) of the participants at the pre-enrolment screening.

The observed lack of RBC alloimmunization reported in the current study may be comparable to the results of the previous studies in some SSA countries demonstrating a low rate of alloimmunization. In a cross-sectional study that included 91 participants with various forms of cancer in Kenya, only 0.9% of them had RBC-alloimmunization and 3.1% had autoantibodies 17. In another study on patients with a wide range of diagnoses which included 108 participants with cancer in Mulago hospital, Natukunda et al. reported RBC alloimmunization in only 13 of the 214 participants (6.1%). Eleven of the 13 participants (85%) with RBC alloimmunization in that study were transfused with 10 or more units of blood 18. However, only 13.3% of participants in our study were transfused with 10 or more units of blood prior to enrolment and only a small number were transfused with 10 or more units of blood during the study. Multiple transfusion episodes is an established risk factor for developing RBC alloimmunization 8. This might explain the disparity of our observation with that of the study by Natukunda et al. 18 The lack of RBC allo-immunization in our study may be due to the possible impaired immune function in patients with cancer, especially patients with lymphoproliferative disorders who are generally known to have an impaired immune response 20. Absent or low RBC allo-immunization rates have also been reported in some multiply transfused patients with HIV infection who have zidovudine (AZT)-associated anaemia and patients with HIV receiving D positive RBC transfusions 21, 22. A proportion of participants with concomitant HIV in the current study was 9.4%. Additionally, unlike in settings with a more cosmopolitan and diverse population, the low RBC allo-immunization rate in our study may also be attributed to the donors and recipients having similar racial antigenic homogeneity 23. It has also been reported that increased age is associated with developing RBC allo-immunization 8. The median age for our study population was 40 years, suggesting that our relatively younger patient population may have impacted the RBC alloimmunization rate.

Four participants with pan reacting antibodies on screening in the current study were not randomized but were included to inform prevalence of RBC allo-antibodies. Further testing with antibody elution or auto adsorption techniques were not possible on the samples from these patients. However, possible explanations for the pan reacting antibodies may be related to their diagnoses and medication exposures. Two of these patients had chronic lymphocytic leukemia that is known to be associated with autoimmune haemolytic anaemia presenting with positive 24; the other patients were exposed to chemotherapy that are known to cause immune mediated haemolytic anaemia 25, 26.

The current study adds to the existing body of knowledge showing that RBC allo-immunization is low in sub-Saharan Africa, especially in patients with cancers. This study is based on a larger number of patients with cancer in comparison to previous studies 17, 18. Additionally, the study examined RBC allo-immunization in a subsection of patients transfused with leucoreduced whole blood that had never been described in the SSA. Leucoreduced blood has been observed to reduce RBC alloimmunization 19. However, with the lack of RBC alloimmunization in our study population, this observation may have not been adequately examined. Some limitations in our study include a significant number of patients being lost to follow up for antibody testing and the likely lack of power. Given the primary aim of the randomized controlled trial was not to assess the efficacy of leucoreduced blood on RBC alloimmunzation, an appropriate sample size to power this outcome was not performed.

Therefore, while it is the recommendation by most blood banks to perform routine RBC allo-antibody screening on all patients requiring blood transfusion, our study suggests that routine RBC allo-antibody screening in patients with cancer in Uganda may require further research.

## Data Availability

All relevant data are within the paper and its Supporting Information file.
